# Fly Ash-Incorporated Polystyrene Nanofiber Membrane as a Fire-Retardant Material: Valorization of Discarded Materials

**DOI:** 10.3390/nano12213811

**Published:** 2022-10-28

**Authors:** Mira Park, Yun-Su Kuk, Oh Hoon Kwon, Jiwan Acharya, Gunendra Prasad Ojha, Jae-Kyoung Ko, Ha-Sung Kong, Bishweshwar Pant

**Affiliations:** 1 Carbon Composite Energy Nanomaterials Research Center, Woosuk University, Wanju 55338, Korea; 2 Woosuk Institute of Smart Convergence Life Care (WSCLC), Woosuk University, Wanju 55338, Korea; 3 Department of Fire Protection and Disaster Prevention, Woosuk University, Wanju 55338, Korea; 4 Convergence Research Division, Korea Carbon Industry Promotion Agency (KCARBON), Jeonju 54853, Korea; 5 Research and Development Division, Korea Institute of Convergence Textile, Iksan 54588, Korea

**Keywords:** electrospinning, polystyrene, fly-ash, waste materials, fire-retardant

## Abstract

Reusing or recycling waste into new useful materials is essential for environmental protection. Herein, we used discarded polystyrene (PS) and fly-ash (FA) particles and a fabricated fly-ash incorporated polystyrene fiber (FA/PS fiber) composite. The electrospinning process produced continuous PS fibers with a good distribution of FA particles. The prepared nanofibers were characterized by state-of-the-art techniques. The performances of the composite nanofibers were tested for fire-retardant applications. We observed that the incorporation of FA particles into the PS fibers led to an improvement in the performance of the composite as compared to the pristine PS fibers. This study showed an important strategy in using waste materials to produce functional nanofibers through an economical procedure. We believe that the strategy presented in this paper can be extended to other waste materials for obtaining nanofiber membranes for various environmental applications.

## 1. Introduction

In recent years, environmental pollution due to several types of waste materials coming from various industrial activities has caused serious problems for the Earth’s entire living population. Every year, tons of new industrial compounds are produced, which are sometimes released into ecosystems. Plastic waste has especially caused enormous environmental pollution because of its non-degradability under natural conditions [1]. Among the highly used plastic materials, polystyrene (PS), one of the promising thermoplastic polymers, is known for its high insulation properties [2]. It has been extensively explored in a variety of applications such as household applications, packaging, consumer electronic products, building and construction, and oil–water separation [3,4,5,6,7,8]. The outstanding features of PS, such as being odorless, colorless, low density, having thermal stability, and its inherent hydrophobic property, make it a promising candidate for various commercial products [9]. Due to the presence of phenyl groups and carbon–carbon (C–C) bonds, polystyrene is highly rigid and resistant to decomposition [10]. Therefore, it is difficult to recycle and reuse PS. Additionally, the recycling process is costly, and there is a very low economic return [11].

Fly ash (FA), a by-product of thermal power plants, is partly used in concrete and cement manufacturing, whereas a large quantity of it is disposed of in landfills [12]. The huge production of FA is extremely worrying because it may cause air and water pollution if it is not disposed of properly [13]. It is primarily composed of several metal oxides such as silica (SiO_2_), alumina (Al_2_O_3_), calcium oxide (CaO), magnesium oxide (MgO), iron oxide (Fe_2_O_3_), titania (TiO_2_), etc. [13,14]. Recently, several investigations have been performed to exploit FA for other applications such as water purification, filtration, adsorption, etc. [13,14,15,16]. Since FA possesses excellent adsorption properties, it has been used as an adsorbent for the removal of organic pollutants and heavy metal ions from water [13,17,18,19].

Over the past decade, there has been a growing emphasis on the utilization of waste materials for environmental and energy applications. Great approaches have been made through various strategies to use waste materials efficiently. Fabricating novel architectures from the discarded waste materials by applying the nanotechnological approach may provide a solution for environmental remediation [20,21]. Nanomaterials offer several amazing characteristics that are superior to that of bulk materials. Currently, electrospinning is considered as the best method for producing continuous nanofibers from various polymer solutions for wide areas of applications such as water treatment, biomedicals, fire-retardant, energy storage, etc. [22,23,24,25,26]. In this regard, several research groups have developed PS nanofibers by an electrospinning technique and applied it for various environmental applications [27,28,29,30,31,32].

Although fire has become an important part of human civilization, it is a major hazard to human life, property, and the environment. Besides the fatal heat levels, the toxic gases or chemicals liberated during a fire also affect the respiratory system and can lead to death. Therefore, there is an urgent need to design and develop cheap and environmentally friendly fire-retardant materials. Electrospun nanofiber membranes can be a good choice for designing such a fire-retardant material, as the nanofibers possess several outstanding characteristics such as flexibility, good mechanical properties, high surface area, porosity, etc. Unfortunately, most of the electrospun nanofibers in the pristine form are combustible. Although the introduction of a halogen- or phosphorus-based flame retardant is the common method of improving the flame retardancy of the materials, the limiting factor is the toxicity of the combustion products from a halogen-based flame retardants. A phosphorous-based flame retardant may be required in large amounts, which not only causes difficulties in the electrospinning process, but also affects the mechanical properties of the fibers [33,34,35]. Therefore, there is an urgent need for modifications to obtain flame retardant derivatives. In recent years, few studies have reported on the development of a fire-retardant nanofiber membrane by incorporating other materials such as nanoclay, metal oxide nanoparticles, graphene, carbon nanotubes (CNTs), etc., into nanofibers [36,37,38,39]. For example, Wu et al. synthesized flame retardant nylon-6 fibers containing montmorillonite clay by an electrospinning technique [40]. Pethsangave et al. prepared phosphorus-functionalized polyaniline and polypyrrole-supported graphene nanocomposites, which showed excellent flame-retardant properties when coated with cotton fabric and wood [41]. Recently, Kang et al. developed thermally oxidized polyacrylonitrile/polyvinylpyrrolidone/SiO_2_ nanofibers as an effective flame-retardant membrane [42]. Thus, the addition of nanofillers can be considered as an alternative approach to improve the fire-retardant performance of electrospun nanofiber membranes [42,43,44].

The utilization of discarded materials as a nanofiber precursor in electrospinning not only provides a remedy for environmental pollution, but also is cost-effective since the discarded materials are freely available [45,46]. Based on these understandings, herein, we used two waste materials (discarded PS and FA particles) to prepare FA-incorporated PS fibers. The as-prepared fiber composite was characterized with various techniques and tested for fire-retardant performance. The strategy presented in this work provides a new approach to using discarded materials for various applications such as a fire-retardant, food packaging, filtration, etc.

## 2. Experimental

### 2.1. Materials

The discarded polystyrene (PS) was collected from the Woosuk University area in Samnye, Jeollabuk-do, South Korea. N,N-Dimethylformamide (DMF) was obtained from Sigma-Aldrich. Purified FA particles were obtained from the Won Engineering Company Ltd., Gunsan, Korea.

### 2.2. Fabrication of FA NPs Incorporated PS Fibers

**Figure 1:**
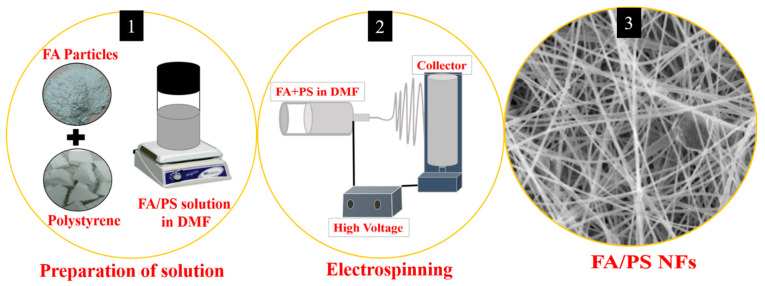
Schematic diagram showing the fabrication of the FA/PS fiber composite.

In the beginning, the FA particles were ball-milled by using 3 mm zirconia balls and sieved for 12 h. A certain amount of ball-milled FA particle was dispersed into the DMF and subjected to sonication for 6 h followed by stirring for 6 h at room temperature. Next, 4 g of discarded PS was added to the above suspension and stirred overnight. The concentration of PS was adjusted to 20%. After obtaining a clear solution, electrospinning was performed at room temperature. During the electrospinning, 20 kV was applied, and the tip-to-collector distance was maintained at 15 cm. The as-spun nanofiber mat was vacuum dried at 40 °C for 12 h. For comparison, a pristine PS fiber mat was also prepared in the aforementioned conditions without using FA. The obtained nanofiber mats were characterized with various techniques. Figure 1 represents the synthetic protocol for the fabrication of the FA/PS fiber composite.

### 2.3. Characterization

The morphologies of the as-prepared nanofibers were investigated by field-emission scanning electron microscopy (FESEM, S-7400, Hitachi, Tokyo, Japan). The average diameter of the nanofibers was determined by using ImageJ software (National Institute of Health, Bethesda, MD, USA). The conductivity of the electrospinning solutions was recorded with the Electrical conductivity meter (CM-42X, DKK-TOA Corporation, Tokyo, Japan). For crystallinity determination, an X-ray diffractometer (XRD) was used (Rigaku Co., Tokyo, Japan). The Fourier transform infrared (FTIR) spectra were recorded using an ABB Bomen MB100 Spectrometer (Bomen, Quebec, QC, Canada). Mechanical properties were studied by using a Universal Testing Machine (AG-5000G, Shimadzu, Kyoto, Japan). The thermal properties of the as-synthesized nanofibers were studied by thermogravimetric analysis (TGA, Perkin-Elmer, Akron, OH, USA).

### 2.4. Evaluation of Fire-Resistance Property

The limited oxygen index (LOI) method was employed to study the fire-retardant property of the as-prepared nanocomposite fibers. The LOI value of the fibers was tested by an M606B digital oxygen index instrument.

## 3. Results and Discussion

**Figure 2:**
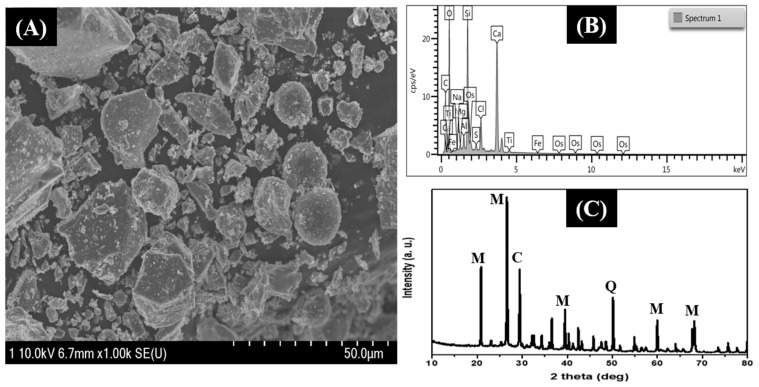
FESEM (**A**), EDS (**B**), XRD spectra (**C**) of FA particles.

The morphological investigation was performed by field emission scanning electron microscopy (FE-SEM). Figure 2A depicts the morphology of ball-milled FA particles. FA is a heterogenous material that consists of small spheres of irregular, porous, coke-like particles. As in Figure 2A, the FA particles were irregular-shaped with various sizes. The chemical composition of the FA particles was confirmed by the EDS analysis. The EDS spectra of FA particles showed the presence of C, O, Mg, Al, Si, S, Cl, Ca, Ti, and Fe (Figure 2B). The XRD spectra identified mullite, calcite, and quartz as the major mineral constituents in FA (Figure 2C) [13,47].

**Figure 3:**
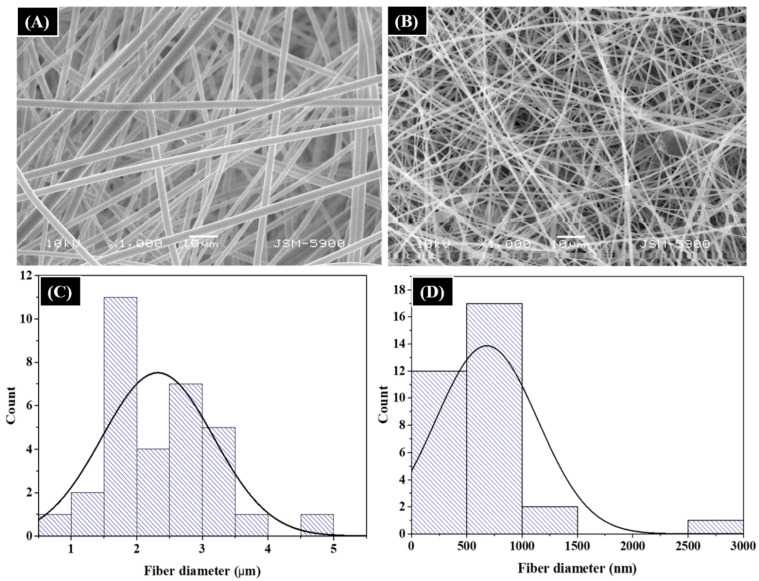
SEM images of as-spun pristine PS fibers (**A**) and FA/PS fibers (**B**). (**C**,**D**) are the diameter distribution curves of PS fibers and FA/PS fibers, respectively.

The morphologies of electrospun PS fibers and the FA/PS composite are given in Figure 3A and Figure 3B, respectively. As in the figures, both samples showed a smooth and continuous fiber morphology without beads. The pristine PS solution resulted in the formation of the microfiber after electrospinning. The average diameter of the pristine PS fibers was 2–3 μm (Figure 3C). Interestingly, the diameter of the PS fibers was reduced to nanoscale after the addition of FA particles. The average diameter of FA/PS fibers was about 600–700 nm (Figure 3D). The nanoscale modification of fibers not only provides a higher surface area, but also widens its application to various fields. Many factors, such as solution concentration, applied voltage, flow rate, tip-to-collector distance, and solution properties (polarity, viscosity, conductivity), influence the diameters and morphology of the electrospun nanofibers [22]. The high reduction of the diameter in the case of composite fibers was attributed to the conductivity of the solution. The conductivity of the PS solution was approximately 2.01 mS/m, whereas it was increased to 2.66 mS/m after the addition of FS particles on it. The increase in the conductivity is attributed to the various metal oxide components of FA. It is well-known that higher conductivity results in the formation of thinner nanofibers. We have observed the same results in our previous studies also [48,49]. The FA/PS composite fibers showed a homogenous distribution of FA particles throughout the nanofibers.

**Figure 4:**
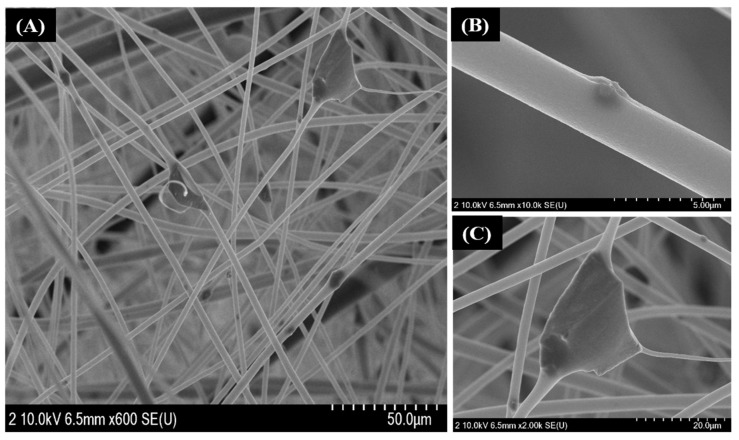
FESEM image of FA/PS NFs membrane (**A**), FA particle embedded inside the PS NFs (**B**), and FA flake-like structure attached to the PS fibers (**C**).

To investigate the arrangement of FA particles in PS fibers, we captured FE-SEM images of the FA/PS composite (Figure 4). As in the figure, the FA particles were both attached to the surface of PS fibers and embedded within the fibers. It should be noted that the FA particles used in this study were in the nano to micro range (Figure 2A). Since the particles were well-dispersed in the PS solution prior to the electrospinning process, the nano-sized particles could be trapped inside the electrospun PS fibers (Figure 4B), while the larger particles were attached to the outer side of the fibers. We also observed a flat-structured FA flake attached to the PS fibers (Figure 4C). Although the size of the FA flake was larger than the diameter of the PS fibers, the flake was attached to the fibers with three connection points, indicating a strong bond with the fibers. This finding ensures the good dispersion of FA particles in PS solution and their interaction with the polymer nanofibers.

**Figure 5:**
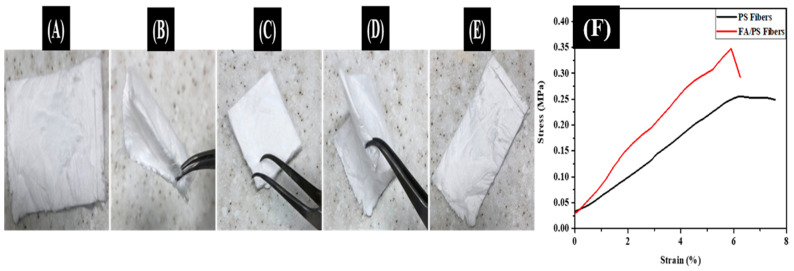
(**A**) Optical images of as-prepared FA/PS membrane, (**B**) during folding, (**C**) folded with 180°, (**D**) during unfolding, and (**E**) after unfolding. (**F**) is the stress–strain curve of the as-prepared FA/PS fiber membrane compared to PS fiber membrane.

The flexibility and the free-standing nature of the FA/PS composite membrane were studied with the folding test. The optical images of the as-prepared FA/PS fiber membrane during the folding tests are given in Figure 5A–E. As in the figure, the product could be folded repeatedly without any damage. Furthermore, the fiber morphology was investigated after being folded with an angle of 180° for one hundred times. There was no change in the fiber morphology. The fibers were preserved intact and showed continuous morphology even after the bending tests several times. The mechanical properties of the as-prepared FA/PS composite fiber membrane were compared with that of the pristine PS fiber membrane. As in Figure 4F, the composite resulted in improved mechanical properties compared to the pristine form. The tensile strength of ~0.23 MPa was recorded in the case of the PS fiber membrane, whereas the FA/PS composite fiber membrane exhibited ~0.34 MPa. The enhancement in the tensile strength is attributed to the role of FA particles in the fiber. The proper interaction between FA and polymer matrix (filler–polymer interaction) is important for a strong nanofiber membrane. The addition of FA particles alters the morphology of the nanofiber membrane. The diameter of the nanofiber was reduced from micro to nanoscale when FA particles were added to the system. The distribution of FA particles in the nano-scaled fibers can substantially improve the mechanical properties of the FA/PS composite fibers due to their large interfacial area, which enables them to transfer an applied load through the filler (or matrix) interface [50,51].

**Figure 6:**
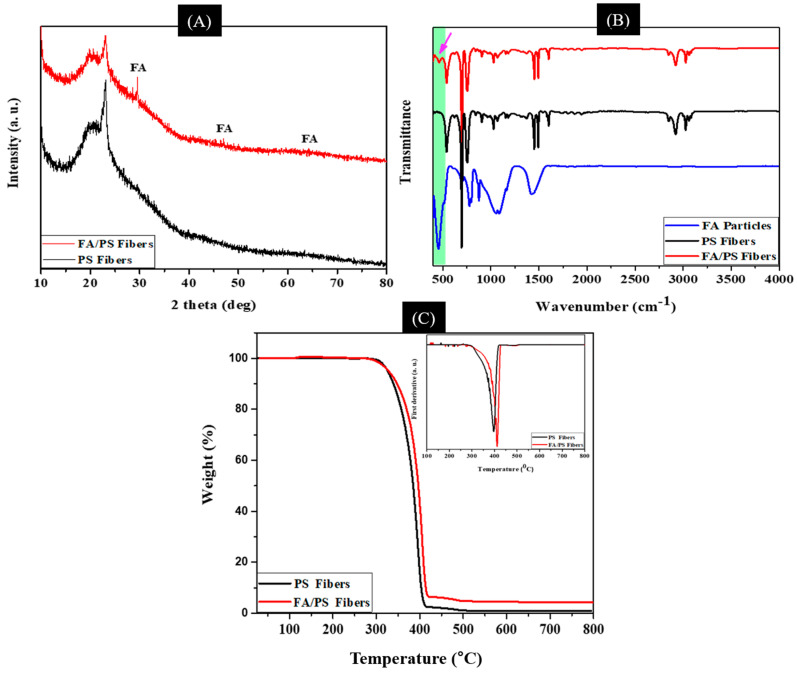
XRD (**A**), FTIR (**B**), and TGA (**C**) of as-prepared samples. The inset in C represents the DTG curves of FA/PS composite membrane compared to that of the pristine PS fibers.

The crystal structure of as-prepared pristine PS and FA/PS fibers was evaluated by an XRD analysis. As shown in Figure 6A, the pristine PS showed two diffraction peaks approximately at 2θ value of 19.1 and 22°, which represents the (110) and (220) crystal planes, respectively [52,53]. For the FA/PS composite, the extra peaks were observed, which confirms the presence of FA particles on it [13]. FTIR spectra of electrospun PS NFs and FA/PS composite are presented in Figure 6B. In the IR spectra of FA, the marked peaks at about 460, 1098, and 1450 cm^−1^ are attributed to the Si–O–Al, Si–O–Si, H–O–H bands, respectively [47,54]. Pure PS showed three obvious characteristic absorption bands. The lower absorbance intensities at 3200–2800 cm^−1^ were assigned as C–H symmetric and asymmetric vibration. The wavenumber at 1600–1400 cm^−1^ corresponds to the bending vibration, and the stronger intensities around 770–650 cm^−1^ are attributed to the mono-substituted benzene [53]. The FA/PS composite showed almost similar peaks as pristine PS. An extra band near 460 cm^−1^ in FA/PS fibers (arrow in Figure 6B) is attributed to the FA in the composite sample [13]. The peak intensity in the FA/PS fibers is remarkably decreased compared to the pristine FA and PS, suggesting the homogenous deposition of FA into PS throughout the matrix [55].

The thermal stability of the prepared samples was studied by thermogravimetric analysis (TGA). The TGA results in Figure 6C provide quantitative information on the incorporation of FA on PS fibers. The TGA profile showed a single-step decomposition in both cases. The degradation occurred around 330–418 °C. The pristine PS fibers showed a complete degradation around 500 °C; however, the FA/PS fibers showed about 6% residue, which is due to the presence of FA in the composite membrane. Additionally, the enhanced thermal property of the composite was verified by the DTG curves (Inset Figure 6C).

**Figure 7:**
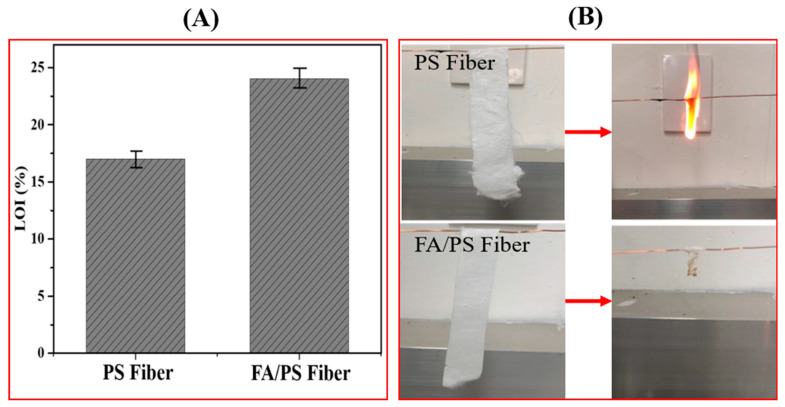
Comparison between the LOI values (**A**) and flame test for PS fiber and FA/PS fiber membrane (**B**).

The limited oxygen index is considered as an excellent tool to quantify the flammability of polymeric materials. The LOI values for pristine PS fibers and FA/PS composite fibers were tested and are given in Figure 7. The PS fibers easily burn in air with black smoke. The limited oxygen index (LOI) value of the pristine PS NFs membrane was recorded at approximately 17%. When FA particles were added to the PS membrane, the membrane possessed an LOI value of ~24%. Further, we carried out an open flame test to access the flame-resistance of the as-prepared membranes (Figure 7B). For this study, the fiber membranes were cut into 1.5 cm × 8 cm and ignited into the air. The pristine PS membrane was ignited immediately with smoke when exposed to the fire source and burnt completely in 3 s. On the other hand, after removing from the fire source, the FA/PS composite membrane shrunk and self-extinguished in about 4 s, without a complete burn. This indicates the role of FA particles in the fire-retardant ability of the composite nanofiber membrane. Recently, Ma et. al. [56] have confirmed that active ingredients (such as Al^3+^, Fe^3+^, Ti^4+^, Mg^2+^, Ca^2+^, etc.) in FA can be released and transformed into effective components of fire prevention. The Fe_2_O_3_ present in FA forms a dense char layer by promoting the crosslinking of the polymer [57]. The strong interaction between FA particles and PS nanofibers promotes the heat resistance of the composite membrane, thereby enhancing the overall fire-retardant properties [58,59].

## 4. Conclusions

The utilization of waste materials such as discarded polystyrene and fly ash particles for designing fire-retardant materials has been introduced in this study. An FA-incorporated PS nanofiber membrane was synthesized by simply dispersing the FA particles in the PS solution followed by the electrospinning process. The as-obtained FA/PS composite showed good nanofiber morphology, along with good distribution of FA particles over the fibers. The performance of the samples was tested for fire-retardant properties, and it was observed that the fire-retardant behavior was enhanced after the addition of FA particles to the PS fibers. The strategy presented in this study highlights the utilization of waste materials, thereby addressing environmental issues. The major attributes of our study are the easy synthetic protocol at ambient conditions and finding out the possibilities of waste-based products in fire-retardant applications. Since the FA particles were well-embedded and attached to the surface of PS fibers, we believe that the as-obtained FA/PS fiber composite can also be applied to various other applications such as food packaging, photocatalysis, adsorption, filtration, etc.

## Data Availability

The data presented in this study are available on request from the corresponding author.
